# Research on the influence of pits on the propagation law of explosion shock waves

**DOI:** 10.1038/s41598-024-62603-0

**Published:** 2024-06-19

**Authors:** Shang Fei, Wang Liangquan

**Affiliations:** Nanjing University of Science and Technology, Jiangsu, 210094 Nanjing China

**Keywords:** Shock wave propagation, Pit, Shock wave diffraction, Numerical simulation, Applied physics, Fluid dynamics, Techniques and instrumentation

## Abstract

To study the influence of pits on shock wave propagation and the propagation of shock waves within pits, numerical simulations were used to calculate the distribution of overpressure peak values at the bottom and rear of the pits at 5 depths and 5 explosion center distances. The results indicate that diffraction occurs when the explosion shock wave passes through the edge of the crater; The peak overpressure of the shock wave at the bottom of the pit exhibits a "spoon shaped" distribution, and the peak overpressure on the right side is significantly higher than that on the left side; There are two distinct boundary regions for the overpressure of the shock wave behind the crater due to the influence of the crater; The distance between the explosion centers has little effect on the distribution trend of the overpressure peak of the shock wave at the bottom and rear of the pit, mainly affecting the magnitude of the overpressure peak. The research results provide theoretical support for the analysis of the propagation law of explosion shock waves and guidance for the design of protective engineering structures, with significant engineering application value.

## Introduction

Explosion shock wave is one of the main factors causing destructive effects on obstacles caused by conventional ammunition explosions. In most cases, the destructive effect of explosion shock waves is caused by overpressure, with overpressure peaks reaching several or even dozens of atmospheres. Previous research on explosive shock waves often focused on open and flat ground, which was relatively ideal. However, the actual environment was often much more complex, with obstacles such as mounds, pits, and trees more or less affecting the propagation of shock waves. Therefore, studying the propagation and distribution of shock waves in typical environments has more practical reference significance.

There has been relevant research both domestically and internationally on the impact of obstacles such as walls on shock wave propagation. Wang Fei et al.^1^ studied the factors that affect the wave blocking effect of retaining walls; Mu^2^, Zhang^3^ and others studied the reflection and diffraction of shock waves after encountering a retaining wall. Zhang^4^ also reached similar conclusions and discovered the relationship between the peak overpressure on the front of the explosion-proof wall and its height through experiments; Li^5^ and Liang^6^ analyzed the distribution of reflected overpressure along the retaining wall at different specific distances; Hong Wu^7^ studied the influence of inclined walls on shock wave propagation. Shi^8^ studied experimentally the factors that affect the weakening of shock waves by spherical obstacles; Shachar Berger^9^, Sha et al.^10^ studied the influence of obstacle geometry and number on shock waves; Chaudhuri^11^ studied the influence of obstacle shape and arrangement on shock wave attenuation. When obstacles act as obstacles to block shock waves, the impact generated by the reflection of the shock wave is greater than that generated by diffraction. However, when the shock wave encounters a pit, the diffraction wave will have a greater impact. Regarding the diffraction of shock waves at edges and corners, Beric Skews^12,13^ studied the diffraction of shock waves at complex convex and circular corners, and predicted the position of diffraction waves at different incident Mach numbers; Gnani^14^ studied the diffraction of shock waves when encountering sharp wedge and circular arc corners. Xu^15^ studied the influence of trench terrain on shock wave propagation, where the width of the trench affects the first peak pressure at the bottom; The peak pressure at the bottom of the trench will decrease with increasing depth, and the impact of depth is minimal when the depth exceeds 0.6 m. However, due to the limited number of measurement points, it only conducted a macroscopic comparison of the shock wave pressure and impulse inside and outside the trench, and did not conduct a detailed study of the distribution patterns inside and behind the trench. At present, there is relatively little research on the impact of pits on shock wave propagation both domestically and internationally. This article analyzes the influence of pit depth on the peak overpressure of shock waves at the bottom and behind the pits through numerical simulation, and studies the range of influence of pits on the area behind them.

## Theoretical analysis of shock wave distribution in pits

**Figure 1:**
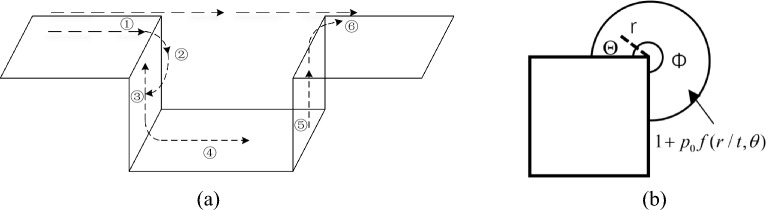
Schematic diagram of shock wave propagation structure in pits. (**a**) Propagation process of shock waves along pits, (**b**) Diffraction wave formation process.

After encountering obstacles during the movement of explosive shock waves in the air, a portion of the shock waves will bypass the obstacles and propagate^16^. According to the principle of Fresnel diffraction, when a wave diffracts an object, the wavefront will bend and diffuse. This is because different points on the wavefront have varying degrees of impact on obstacles. Near the edge of the obstacle, the wavefront is restricted, while behind the obstacle, the wavefront begins to expand again, forming diffraction waves. According to aerodynamics theory, a two-dimensional inviscid supersonic airflow passes through a bend angle of θ When the outer convex angle is increased, the flow space expands, the airflow expands, and the pressure and temperature decrease. The disturbance of pressure changes propagates outward from the convex corner, forming a weak expansion wave. When the airflow passes through a continuous convex angle, each point in the curved part will emit a weak expansion wave, Forming a "continuous expansion wave"^17^. Boundary layer separation is also prone to occur near convex corners. Due to the viscosity of the air itself, there is a boundary layer between the air and the wall. Under the action of the inverse pressure gradient, boundary layer separation occurs, and the separated boundary layer will be mixed with the main flow, affecting the pressure of the main flow. The propagation law of shock waves in pits is shown in Fig. 1.

According to this principle, the process of shock waves from ① to ② is equivalent to passing through countless externally folded micro element walls, and ultimately the pressure of the shock wave follows the curve during propagation angular propagation changes the direction of propagation, and the shock wave moves along the front wall to form a diffracted wave after the incident wave. Takano^18^ believes that the pressure in the circular sector where the shock wave of pressure P_0_ propagates along the corner can be expressed as $1 + p_{0} f(r/t,\theta )$, where:

1 $$ f(r/t,\theta ) = \frac{1}{\pi }{\text{Tan}}^{ - 1} \left[ {\frac{{(1 - R^{2} )\sin \alpha }}{{(1 + R^{2} )\cos \alpha - 2R\cos \omega }}} \right] $$

In the equation, $R = \rho^{\lambda } ,\omega = \lambda \theta ,\alpha = \lambda \pi ,\lambda = \pi /\Phi_{1}$. When $\phi_{1} = 3/2\pi$, the distribution of scattered waves from ① to ② can be obtained.

Subsequently, the diffraction wave and the original waveform are superimposed and continue to propagate, and reflection occurs at point ③. After being superimposed again, it continues to propagate along the wall. Some shock waves propagate along the bottom of the pit from ③ to ④, and multiple reflections occur during the process.

In the process of diffraction wave propagation along the concave pit, if the incident angle exceeds a certain limit angle $\alpha_{0\max }$. When incident at $\alpha_{0}$, the diffraction wave is used as the incident wave, which combines with the reflected wave generated by the concave ground to form a new shock wave, known as the "Mach wave". In the common mode of Mach wave formation (explosive explosion), Mach wave peak overpressure

2 $$ \Delta {\text{P}}_{M} { = }\Delta {\text{P}}_{{0}} (1 + \cos \alpha_{0} ) $$

In the formula, $\Delta P_{0}$ represents the peak overpressure during ground explosions with the same amount of charge;

During the process from ④ to ⑤, the front of the bottom shock wave collides with the right side wall of the pit, causing a positive reflection of the wave. The overpressure on the wall suddenly increases to the reflected overpressure, causing an increase in the peak overpressure at the bottom right side of the pit. The overpressure reflected by air shock waves on a rigid surface can be expressed as

3 $$ \vartriangle p_{n} = 2p_{m} + \frac{\gamma + 1}{2}\rho_{m} u_{m}^{2} $$

In the formula, $\gamma$ is the air adiabatic index, and m represents the parameters of the shock wave front. This process is equivalent to the interaction between two equally strong colliding shock waves, with the first term on the right considering the sum of the overpressure components (static pressure components) of the two interacting waves, and the second term considering the dynamic components caused by velocity flow braking. After a certain period of time, a standing pressure is established on the right wall of the pit.

Similarly, during the propagation process from ⑤ to ⑥, there is also diffraction phenomenon. The shock wave moving upwards along the wall will overlap with the shock wave passing directly above, causing the shock wave pressure at ⑥ behind the pit to rise.

## Simulation analysis of the impact of pits on the overpressure distribution of shock waves

### Material parameters and simulation model

**Figure 2:**
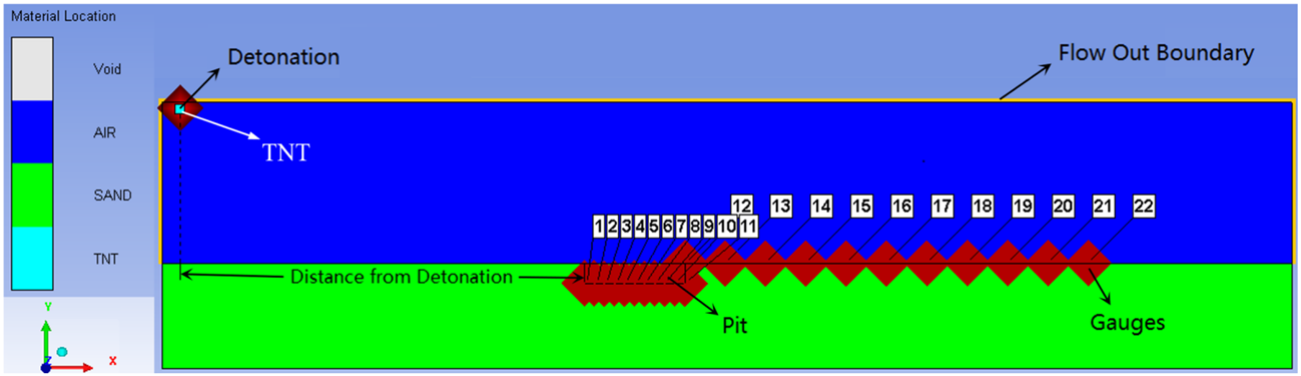
Numerical simulation model of explosion shock wave.

This study used the explosion display dynamics simulation software AUTODYN to conduct numerical simulation analysis of the distribution law of shock wave pressure propagation^19^. Based on the fact that the actual testing in the shooting range is not a plan, a 1:1 finite element numerical simulation model is established, and the constructed model is shown in Fig. 2. The model mainly consists of three parts, namely air, sand, and TNT explosives. Below the model is a sandy ground. Dig a pit on the sandy ground and fill the rest with air, which is an ideal gas. Explosives are added to the air by filling. The size of the sand ground structure is 5600 mm × 540 mm × 520 mm, with a grid size of 5 mm × 5 mm × 5 mm, with an air domain structure size of 5600 mm × 540 mm × 800 mm, with a grid size of 2 mm × 2 mm × 2 mm, the explosive is 750 mm above the ground, and the grid size is 1 mm^20,21^. In order to simulate the semi infinite air domain environment in actual explosion environments, the boundary conditions of the air domain module on all three sides except for the axis of symmetry are set as pressure outflow. The parameters of the pits in the model are shown in Table 1. During the simulation, 11 measuring points were arranged in the pit with a spacing of 50 mm, and 12 measuring points were arranged behind the pit with a spacing of 200 mm. The horizontal distance between the measuring point and the perpendicular projection point of the explosion center is called burst distance^22,23^.

**Table 1:** Pit structure parameters.

Length (mm)	Width (mm)	Burst distance (mm)	Depth (mm)
500	250	2000	100, 200, 300, 400, 500
500	250	2250	100, 200, 300,400, 500
500	250	2500	100, 200, 300, 400, 500
500	250	2750	100, 200, 300, 400, 500
500	250	3000	100, 200, 300, 400, 500

In the above model, air is described by Ideal Gas equation of state, which is expressed as follows^24^:

4 $$ P = E \cdot (\gamma - 1) \cdot \rho /\rho_{0} $$

where $P$ is the gas pressure; $\gamma$ is the parameter of ideal gas equation of state; $\rho$ is the air density; $\rho_{0}$ is the initial gas density; $E$ is the energy density (explosive energy per unit volume). The parameters are shown in Table 2.

**Table 2:** Main property parameters of air materials.

$\rho_{0} \left( {{\text{kg}}\;{\text{m}}^{ - 3} } \right)$	$\gamma$	$E\left( {{\text{MPa}}} \right)$
1.225	1.4	0.2533

The JWL equation of state is adopted for TNT explosive, and its equation is as follows^25^:

5 $$ P{\text{ = A}}\left( {1 - \frac{\omega }{{R_{1} V}}} \right)e^{{ - R_{1} V}} + B\left( {1 - \frac{\omega }{{R_{2} V}}} \right)e^{{ - R_{2} V}} + \frac{\omega }{V}E{\kern 1pt} $$

In the above formula, $P$ is pressure, $V$ is volume, $E$ is internal energy, $R_{1}$ and $R_{2}$ are material parameters, and $R_{1}$, $R_{2}$ and $\omega$ are constants^26^. The specific values of the parameters are shown in Table 3.

**Table 3:** Parameters of JWL equation of state for TNT explosive.

A (Mbar)	B (Mbar)	R_1_	R_2_	ω
8.807	0.184	4.15	0.9	0.35

SOIL_AND_FOAM_FAILURE material model is selected for sand soil state equation, which has fluid properties in some aspects. The plastic yield limit function $\phi$ is described by the second invariant $J_{2}$ of the stress deviator^27^.

4 $$ \phi = J_{2} - (a_{0} + a_{1} p + a_{2} p) $$

where $J_{2} = S_{ij} /2$; $a_{0} ,a_{1} ,a_{2}$ are a constant; $p$ is the pressure. The main technical index parameters of sandy soil are shown in Table 4.

**Table 4:** Main property parameters of sandy soil material.

$\rho \;({\text{g}}\;{\text{cm}}^{ - 3} )$	$a_{0} \;({\text{GPa}})$	$a_{1} \left( {{\text{GPa}}} \right)$	$a_{2} \left( {{\text{GPa}}} \right)$	$p_{c} \left( {{\text{GPa}}} \right)$
1.8	$3.4{\text{E}}^{ - 13}$	$7.0{\text{E}}^{ - 7}$	0.3	$- 6.9{\text{E}}^{ - 8}$

After the above models are established, the finite element numerical simulation analysis of the shock wave pressure propagation distribution law in the process of ammunition explosion can be carried out by setting the simulation parameters of each part of the AUTODYN simulation software.

### Propagation law of shock waves in pits

**Figure 3:**
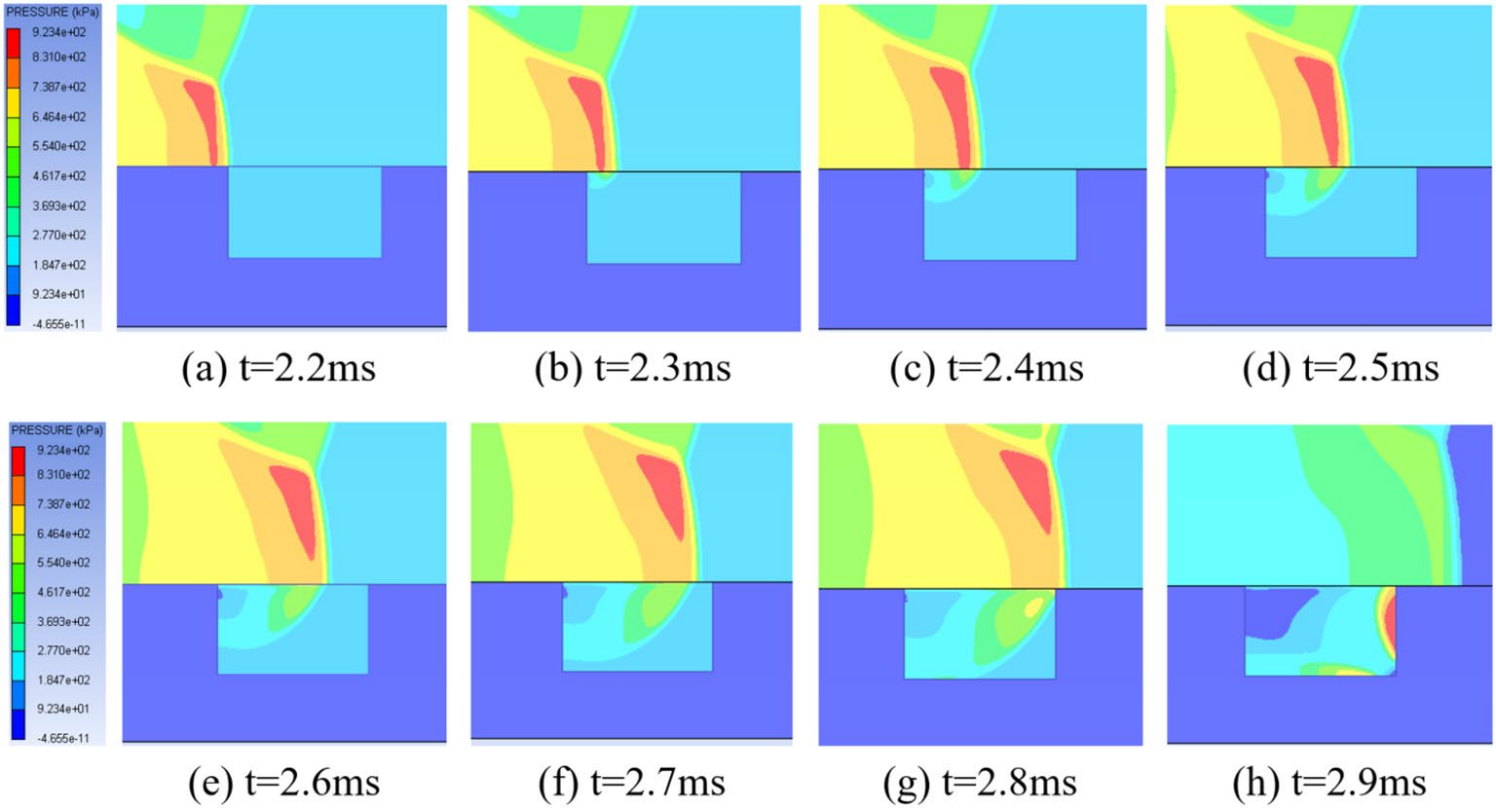
Propagation of shock waves in deep pit of 300 mm.

To study the diffraction of shock waves at the edge of the pit and the propagation process inside the pit, Fig. 3 shows the propagation process of shock waves in a pit with a depth of 300 mm at different times.

From the Fig. 3, it can be seen that the shock wave begins to diffract after passing through the edge of the crater, and obvious diffraction can be seen at t = 2.4 ms. On the one hand, the shock wave above the pit continues to propagate forward, and on the other hand, the diffraction wave in the pit contacts the side wall of the pit and reflects. There is a pressure difference between the reflected wave and the diffraction wave on the wall, causing air to flow from the higher pressure area to the lower pressure area. A portion of the diffracted waves will flow upwards along the left wall of the pit, while the remaining diffracted waves will continue to move downwards and reflect upon contact with the bottom of the pit. After the reflection wave and diffracted wave are superimposed, they will move forward along the bottom of the pit, ultimately touching the right wall of the pit. The pressure of the shock wave also reaches its maximum on the right wall of the pit.

### Distribution of shock wave overpressure at the bottom of the pit

**Figure 4:**
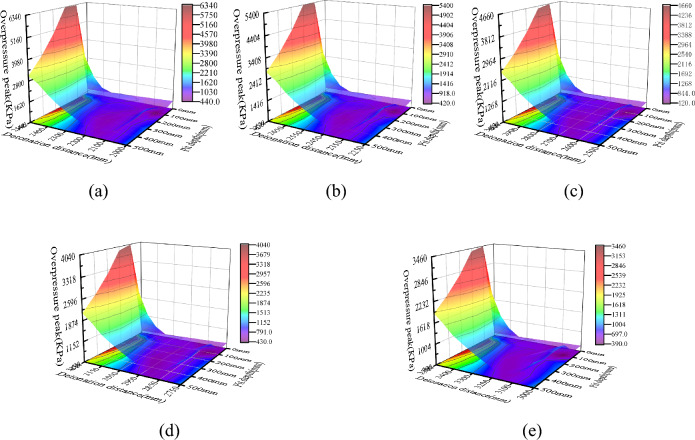
Peak overpressure at the bottom of pits at different depths under 5 different explosion center distances. (**a**) Burst center distance 2000 mm, (**b**) Burst center distance 2250 mm, (**c**) Burst center distance 2500 mm, (**d**) Burst center distance 2750 mm, (**e**) Burst center distance 3000 mm.

The shock wave will diffract after passing through the edge of the pit, and after being reflected by the sidewalls and bottom of the pit, the overpressure of the shock wave inside the pit will undergo significant changes. Figure 4 shows the distribution of peak overpressure at the bottom of pits at different depths under five different detonation center distances. The X-axis in the figure represents the distance between the measuring point and the explosion center, in millimeters; The Y-axis represents the depth of the pit, in millimeters; The Z-axis represents the peak shock wave pressure, measured in kPa. The curve in the XY plane is the contour line of the projection of the peak shock wave pressure on the bottom surface.

From the graph, it can be seen that the peak overpressure of the shock wave at the bottom of the pit presents a spoon shaped distribution of low on the left and high on the right, and the overpressure value on the right is significantly higher than that on the left, which is consistent with the propagation process of the shock wave inside the pit. The peak overpressure near the left wall of the pit bottom (measurement point 1) is 564.96 kPa (explosion center distance 3000 mm), which is 975.48 kPa (explosion center distance 3000 mm) higher than the peak overpressure without pits. Compared with the two, it can be seen that the peak overpressure with pits is 60% to 70% higher than that without pits; The peak overpressure near the right wall of the pit bottom (measuring point 11) is related to the depth of the pit. The peak overpressure without the pit is 464.51 kPa (explosion center distance 3500 mm), and the peak overpressure with the pit is 3376 kPa. Compared with the two, it can be seen that the peak overpressure with the pit is 4–8 times that without the pit.

**Figure 5:**
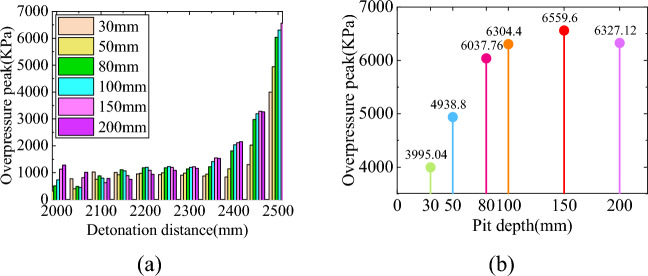
Peak overpressure of shock wave at the bottom of shallow pits. (**a**) Peak overpressure at the bottom of shallow pits, (**b**) Peak overpressure on the right side of pits with different depths.

To further investigate the relationship between the peak overpressure near the right wall of the pit bottom and depth, simulations were conducted on four depths of pits: 30 mm, 50 mm, 80 mm, and 150 mm. The results are shown in Fig. 5a. The relationship between the peak overpressure near the right wall of the pit bottom and the depth of the pit is shown in Fig. 5b. Based on Figs. 4 and 5, it can be concluded that the peak overpressure of the shock wave near the right wall of the pit bottom (measuring point 11) shows a trend of first increasing and then decreasing with increasing depth, and the maximum overpressure peak corresponds to a pit depth between 100 and 200 mm.

**Figure 6:**
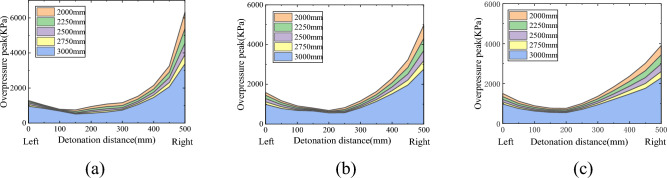
Peak overpressure at the bottom of some deep pits under different explosion center distances. (**a**) Depth 200 mm, (**b**) Depth 300 mm, (**c**) Depth 400 mm.

To more intuitively represent the impact of blast center distance on the peak overpressure of the shock wave at the bottom of the pit, a new coordinate system was established with the left edge of the pit bottom (measuring point 1) as the origin. Under this coordinate system, the peak overpressure of the shock wave at the bottom of each depth pit is shown in Fig. 6. Under different detonation center distances, the variation trend of the peak overpressure of the shock wave at the bottom of the same depth pit is similar, still showing a "spoon shaped" distribution between high, medium, and low on both sides. The detonation center distance mainly affects the peak overpressure of the shock wave. Overall, the peak overpressure of the shock wave inside the pit decreases with increasing blast center distance, but its attenuation rate varies at different positions at the bottom of the pit.

### Overpressure distribution of shock waves behind pits

**Figure 7:**
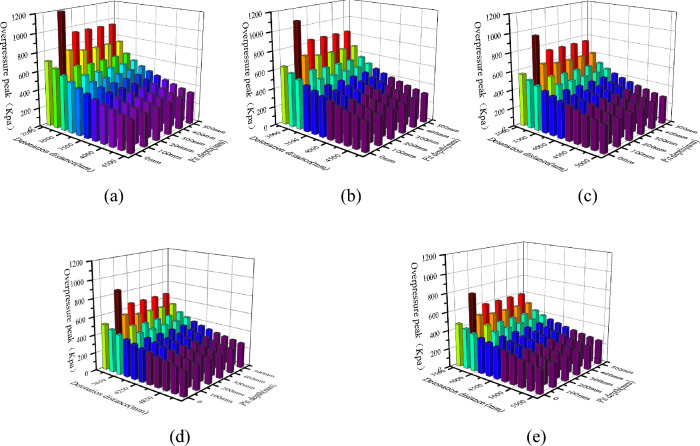
Overpressure peak after different depths of pits under 5 types of explosive center distances. (**a**) Burst center distance 2000 mm, (**b**) Burst center distance 2250 mm, (**c**) Burst center distance 2500 mm, (**d**) Burst center distance 2750 mm, (**e**) Burst center distance 3000 mm.

Due to the diffraction of the shock wave after passing through the pit, the overpressure of the shock wave significantly increases after multiple reflections of the diffraction wave on the pit wall. When the reflected wave moves upwards along the sidewall of the pit, it will affect the overpressure distribution of the shock wave behind the pit. To analyze the influence of pit depth and blast center distance on the distribution of shock wave overpressure behind the pit, Fig. 7 shows the distribution of peak overpressure behind the pit at different depths under five different blast center distances.

**Figure 8:**
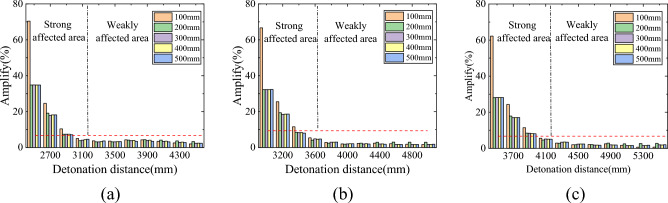
The amplitude of overpressure peak at each depth of pit relative to no pit. (**a**) Burst center distance 2000 mm, (**b**) Burst center distance 2500 mm, (**c**) Burst center distance 3000 mm.

Affected by pits, the peak overpressure behind them is higher than that without pits, and the amplitude of the overpressure peak increases as it approaches the pit. The relative amplitude of the overpressure peak without pits is shown in Fig. 8. As shown in the figure, there are two distinct boundary areas for the pressure behind the pit. Divide the rear of the pit into strong and weak impact areas based on the strength of the affected area. In the strong impact zone, the peak overpressure of the shock wave decays rapidly with increasing distance, and the depth of the pit will have an impact on the peak overpressure of the shock wave behind it, mainly concentrated near the edge of the pit. The overpressure peak at the edge of the 100 mm deep pit is significantly higher than that of the other deep pits, with an increase of 60% to 70% compared to the peak overpressure without pits; The variation trend of overpressure peak in the area with a depth of 200–500 mm is similar, and the increase in overpressure peak at the edge is about 25%. The comparison shows that the impact caused by the depth of the pit is mainly manifested when the depth is shallow, and the main reason for this phenomenon is influenced by the reflected waves on the right wall and bottom of the pit. In the weakly affected zone, the decay rate of the overpressure peak becomes relatively flat, and the influence of the depth change of the pit on the subsequent overpressure peak can be almost ignored. In Fig. 7, the trend of the overpressure peak values behind the pits at various depths is almost consistent in this area. In terms of numerical values, the increase in overpressure peak values relative to the absence of pits is less than 5%, usually between 2 and 3%. Therefore, in the weakly affected area, the influence of pits on the distribution of shock wave overpressure peak values in this area can be ignored.

**Figure 9:**
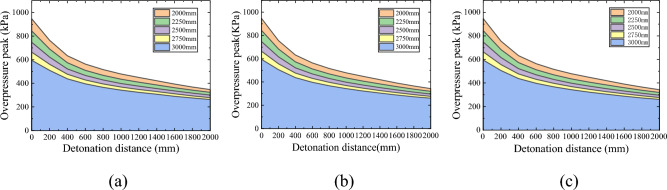
The amplitude of overpressure peak at each depth of pit relative to no pit. (**a**) Depth 200 mm, (**b**) Depth 300 mm, (**c**) Depth 400 mm.

To more intuitively represent the impact of blast center distance on the peak overpressure of the shock wave behind the pit, a coordinate system was re established with the left edge of the ground behind the pit (measuring point 12) as the origin. Under this coordinate system, the peak overpressure of the shock wave behind each depth of the pit is shown in Fig. 9. The variation trend of the peak overpressure of the shock wave behind the same depth pit is similar under different blast center distances, and the blast center distance mainly affects the peak overpressure of the shock wave. The closer the pit is to the center of the explosion, the greater the overpressure of the shock wave behind the pit.

## Experimental study on the influence of pits on the distribution of shock wave overpressure

### Test site layout

**Figure 10:**
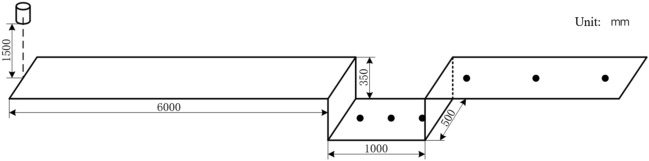
Site layout diagram.

The on-site layout is shown in Fig. 10, with a pit length of 1 m, width of 500 mm, and depth of 350 mm. The left edge of the pit is 6000 mm away from the explosion center. Three measuring points are arranged inside the pit, with distances of 170 mm, 500 mm, and 830 mm respectively from the left wall of the pit; three measuring points were also arranged behind the pit, with distances of 250 mm, 1250 mm, and 2250 mm from the edge of the pit. The TNT is 1500 mm above the ground, with a charge of 8 kg and 16 kg, respectively.

### Composition of testing system

**Figure 11:**
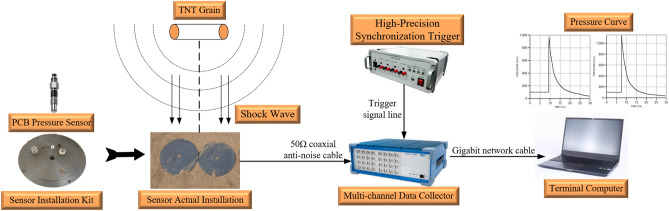
Test system composition.

The composition of the testing system is shown in Fig. 11, consisting of a pressure sensor, sensor mounting base, data acquisition system (including ICP signal conditioning module), upper computer, low noise coaxial cable, and testing software. To ensure the accuracy of the test results, all sensors used in the test have been calibrated.

The composition of the testing system is shown in Fig. 11, consisting of a pressure sensor, sensor mounting base, data acquisition system (including ICP signal conditioning module), upper computer, low noise coaxial cable, and testing software. The 113B27 pressure sensor from PCB company is selected for pressure sensing. The data collection system is the TranNET EPC series data collection system produced by Elsys company in Switzerland. The sampling frequency of the data acquisition system in this experiment is set to 1 MHz; the triggering method is set to software triggering; The data collection method is continuous collection and data flow disk.

**Figure 12:**
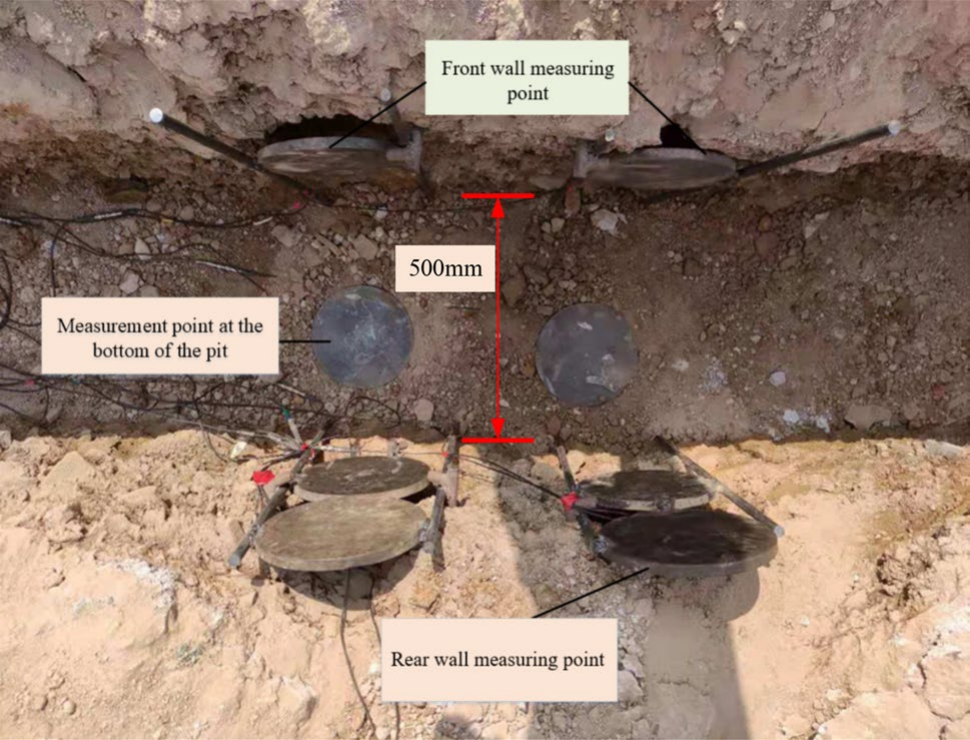
Actual structure of the tunnel.

The on-site layout is shown in Fig. 12, with a pit length of 1000 mm, width of 500 mm, and depth of 350 mm. The left edge of the pit is 6000 mm away from the explosion center. Three measuring points are arranged at the bottom of the pit, with distances of 170 mm, 500 mm, and 830 mm from the left wall of the pit respectively; three measuring points are also arranged behind the pit, with distances of 250 mm, 1250 mm, and 2250 mm from the edge of the pit. The TNT is 1500 mm above the ground, with a charge of 8 kg and 16 kg, respectively.

### Analysis of test results

**Figure 13:**
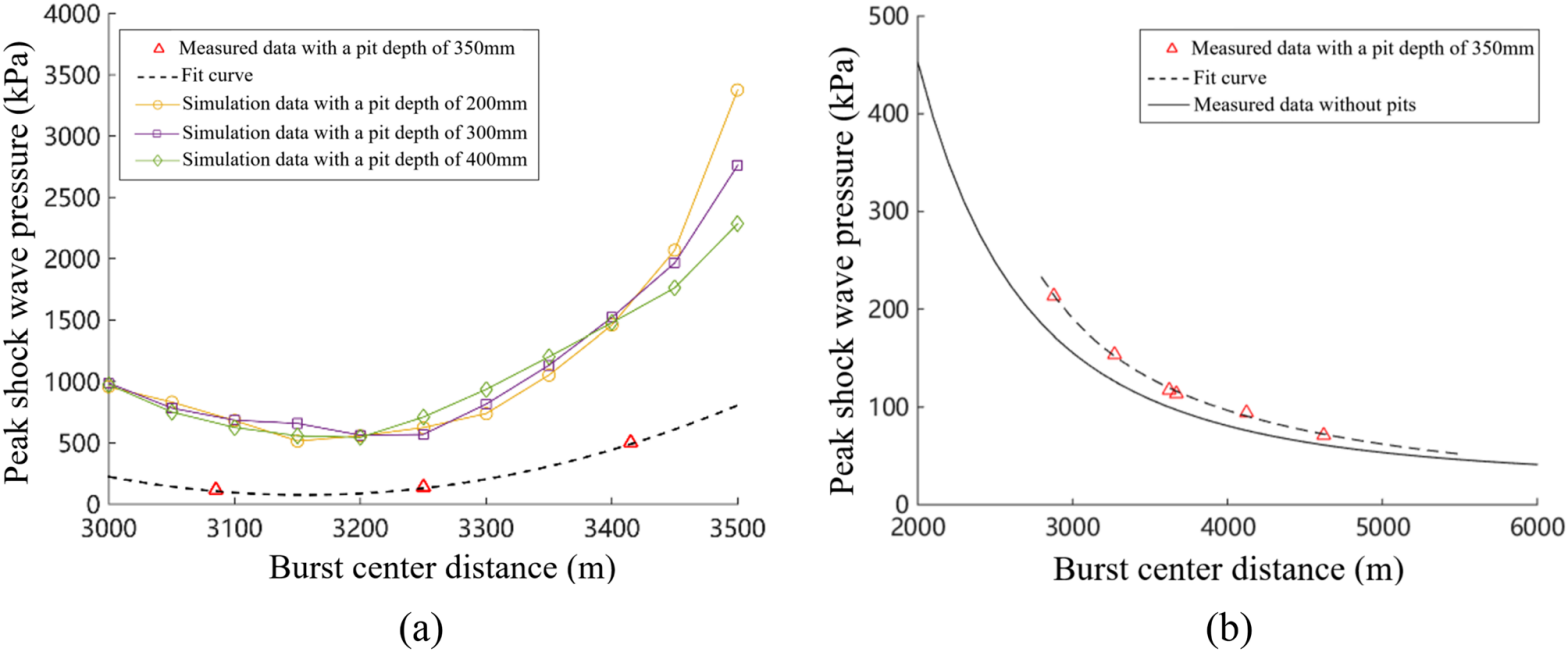
Test results of the impact of pits on the distribution of shock wave overpressure. (**a**) Measured curve of overpressure peak at the bottom of the pit, (**b**) Measured curve of overpressure peak after the pit.

Figure 13 is a curve drawn based on the measured data of a 350 mm deep pit. Due to the large attenuation rate of the sand soil surface during the propagation of shock waves, and the many factors that affect the propagation law of shock wave pressure in actual testing, the measured pressure peaks at the same distance from the explosion center are all smaller than the simulated shock wave pressure peaks. In Fig. 13a, the measured pressure peak at the bottom of a 350 mm deep pit was compared with the simulated pressure peak at a depth of 200–400 mm. The trend of the pressure peak at the bottom of the pit is basically consistent with the trend of the shock wave pressure peak obtained from numerical simulation. The pressure peak at the bottom of the pit shows a spoon shaped distribution, and the pressure peak at the right edge of the pit is greater than that at the left edge. Figure 13b shows the variation law curve of the peak shock wave pressure measured in a 350 mm deep pit. Similarly, it can be concluded that the trend of pressure peak changes after the depression is basically consistent with the law of shock wave pressure peak changes obtained from numerical simulation. When there are pits on the testing site, the peak pressure of the shock wave generally increases compared to when there are no pits, and the pressure peak at the edge of the pits increases by more than 20%; The closer to the edge of the pit, the faster the rate of pressure peak attenuation with distance; As the distance increases, the influence of pits on the pressure peak gradually weakens. After the distance increases to a certain extent, the peak pressure of the shock wave with pits is close to that without pits. From the above analysis, it can be seen that the peak pressure of the shock wave on the left edge of the pit is smaller than that on the right side. Therefore, in the design of protective structures, the safety area for protective personnel needs to be located inside the pit near the explosion center.

## Conclusions

Through numerical simulation analysis of the explosion shock wave passing through different depths of pits, combined with relevant experimental results, the following conclusions were obtained:

1. The shock wave undergoes diffraction after passing through the edge of the crater. The shock wave overpressure at the bottom of the pit presents a "spoon shaped" distribution, and the peak overpressure on the right side is significantly higher than that on the left side. The overpressure peak on the right shows a trend of first increasing and then decreasing as the depth increases, and the maximum overpressure peak corresponds to a pit depth between 100 and 200 mm.
2. The depression will increase the peak overpressure behind it, and there are two distinct boundary areas. In the strong impact zone, shallow pits will significantly increase the peak overpressure of the shock wave at the rear edge; the overpressure peak of the shock wave in the weakly affected zone is less affected by the concave pit.
3. After determining the size of the pit, the distance between the pit and the explosion center has little effect on the distribution trend of the overpressure peak of the shock wave, while the explosion center distance mainly affects the size of the overpressure peak.

## Data Availability

All data generated or analysed during this study are included in this published article [and its supplementary information files].
